# 

**Published:** 1924-09

**Authors:** 


					?
New Series. Vol.111. No. 36 September, 1924. Monthly, Price 6d. (pos7T,5REE)
EDUCATIONAL NUMBER.

				

